# Psychosocial stress and strategies for managing adversity: measuring population resilience in New South Wales, Australia

**DOI:** 10.1186/1478-7954-8-28

**Published:** 2010-10-14

**Authors:** Melanie Taylor, Margo Barr, Garry Stevens, Donald Bryson-Taylor, Kingsley Agho, Jennifer Jacobs, Beverley Raphael

**Affiliations:** 1 School of Medicine, University Western Sydney, Building EV, Parramatta South Campus, Locked Bag 1797, Penrith South DC, NSW, 1797, Australia; 2 NSW Department of Health, Sydney Australia, Locked Mail Bag 961, North Sydney, NSW, 2059, Australia

## Abstract

**Background:**

Populations around the world are facing an increasing number of adversities such as the global financial crisis, terrorism, conflict, and climate change. The aim of this paper was to investigate self-reported strategies and sources of support used to get through "tough times" in an Australian context and to identify patterns of response in the general population and differences in potentially vulnerable subgroups.

**Methods:**

Data were collected through a cross-sectional survey of the New South Wales population in Australia. The final sample consisted of 3,995 New South Wales residents aged 16 years and above who responded to the question: "What are the things that get you through tough times?"

**Results:**

Respondents provided brief comments that were coded into 14 main subject-area categories. The most frequently reported responses were family and self (52%); friends and neighbors (21%); use of positive emotional and philosophical strategies (17%), such as sense of humor, determination, and the belief that things would get better; and religious beliefs (11%). The responses of four population subgroups were compared, based on gender, household income, level of psychological distress, and whether a language other than English was spoken at home. Women reported greater use of friends and neighbors and religious or spiritual beliefs for support, whereas men reported greater use of drinking/smoking and financial supports. Those with lower incomes reported greater reliance on positive emotional and philosophical strategies and on religious or spiritual beliefs. Those with high levels of psychological distress reported greater use of leisure interests and hobbies, drinking/smoking, and less use of positive lifestyle strategies, such as adequate sleep, relaxation, or work/life balance. Those who spoke a language other than English at home were less likely to report relying on self or others (family/friends) or positive emotional and philosophical strategies to get through tough times.

**Conclusions:**

Understanding strategies and sources of support used by the population to get through adversity is the first step toward identifying the best approaches to build and support strengths and reduce vulnerabilities. It is also possible to reflect on how large-scale threats such as pandemics, disasters, conflict, bereavement, and loss could impact individual and population resilience.

## Background

The metaphor "tough times" is used with increasing frequency in the media and in general conversation to describe any of a number of stressors and adversities acting on individuals or the population. Within Australia, the phrases "going through tough times" and "doing it tough" are part of everyday language, phrases that are matter-of-fact and somewhat impassive. Such phrases are popular in the media and are often used by national charities and welfare agencies, including ReachOut Australia, Wesley Mission, and Child Support Agency, as the phrases can be used to engage people across a range of demographics, especially males and people who may be reluctant to seek help and advice. This terminology has also been adopted by Australian researchers working in the field of resilience and men's mental health for similar reasons [1].

For the general population, the phrase "tough times" is a nonthreatening and inclusive phrase that can be used in the context of a wide range of adversities, such as financial hardship, coping with pressures, and mental health concerns as well as wider issues such as working through the global financial crisis, living with the consequences of drought, or dealing with disasters and emergencies, such as floods and bush fires. The widespread use and acceptance of the phrase in the Australian language also promotes a nationally proud and resilient culture that acknowledges that we all encounter adversities but that we adapt and bounce back.

In the context of the current research, resilience is regarded, in general terms, as the capacity for successful adaptation or change in the face of adversity [2], and therefore successfully negotiating adversity is regarded as an indicator of resilience. Coping with and managing adversity and moving on to adapt and maintain functioning are key indicators assessed in population-level resilience research, typically in response to mass adversities, disasters, etc. [3,4]. Bleich et al [3] studied the Israeli population and its response to the prolonged and continuing threat of terrorism and found relatively low levels of psychiatric morbidity (<10% meeting criteria for post-traumatic stress disorder symptoms) in a highly exposed population. They attributed this to a possible combination of coping mechanisms and habituation. Specific coping mechanisms included seeking social support (both emotional and instrumental), religious beliefs, humor and positive emotions, self-distraction, denial, and use of alcohol and cigarettes. In recent disaster research, Norris et al [4] have extended models of population resilience away from a focus on psychopathology and more to focus on resilience trajectories. Like many other researchers, they also note that resilience is the norm in populations facing adversity and that this normative adaptation is to be expected.

A number of researchers have been able to investigate individual and population response to adversities and investigate associations between certain strategies and coping approaches and psychological health. In research before and after the September 11 terrorist attacks in New York, Fredrickson et al [5] found that positive emotions were effective at protecting against depression in resilient individuals and that these emotions helped assist resilient people to thrive. The role of social support in relation to coping with adversity and disaster and to being resilient as communities and individuals has been widely reported in the research literature. Masten [6] refers to "ordinary magic" to encompass the role of ordinary processes in building resilience. This includes relationships with families, friends, and communities and both emotional and practical support. Again, disaster research has included assessment of resource loss following natural disasters and found that low social support and loss of "personal characteristic" resources such as optimism or the sense of life having purpose predicted psychological distress [7].

The current climate has led us to re-examine population data collected in Australia during the first six months of 2007 as part of a series of question modules included in the New South Wales (NSW) Population Health Survey [8,9]. The aim of this paper is to analyze responses to a single open-ended question: "What are the things that get you through tough times?" Identifying and quantifying the key strategies and sources of support that people use to get through tough times is the first step toward enhancing individual and population resilience. We analyze the patterns of approach used by the Australian general population to cope with tough times and identify relative differences in the strategies employed by potentially more vulnerable subgroups: women, those with low household incomes (<AUD$40,000), those with high levels of psychological distress, and those who speak a language other than English at home.

## Methods

The question -- "What are the things that get you through tough times?" -- was included in a series of short question modules incorporated into the NSW Population Health Survey between Jan. 22 and June 30, 2007. These questions were field-tested on a sample of 200 NSW residents to quantify test re-test reliability and ensure ease of understanding and lack of ambiguity before being included in the main survey. This research was approved by the human research ethics committees of the University of Western Sydney and the NSW Department of Health.

The NSW Population Health Survey is an ongoing survey of the health, health behaviors, and service use of people living in NSW. It is administered using computer-assisted telephone interviewing (CATI). The target population for the survey is adult NSW residents aged 16 and older living in households with private telephones stratified by geographic location. Households are sampled using list-assisted random digit dialing, and one person aged 16 years and older from each household is randomly selected for inclusion in the survey. Trained interviewers at the Health Survey Program CATI facility carry out interviews. Up to seven calls are made to establish initial contact with a household, and up to five calls are made in order to contact a selected respondent. Data are collected from approximately 1,000 NSW residents each month. The population survey data are weighted to adjust for probability of selection and for differing nonresponse rates among males and females and different age groups. Further details of the NSW Population Health Survey methodology, sampling procedure, and weighting procedure may be found in Barr et al [10].

### Response coding

The question -- "What are the things that get you through tough times?" -- was an open-ended question, and the telephone interviewers recorded each respondent's answer verbatim. As the context of data collection was within the larger health survey, in which respondents mostly were asked to provide ratings or simple yes/no responses, the responses were typically short, one- or two-word responses -- "my faith." "family," "friends," "going fishing," etc. Occasionally, they were more descriptive -- "positive outlook," "talking to husband/friends," "sense of humor," for example. As there was very little detail and no discursive comment data recorded, the categorization of these qualitative data was straightforward. Most comments were discrete and unambiguous, although they covered a wide range of emotional, practical, and symptom-related strategies and also included the use of a variety of resources, such as significant social support (family, friends, neighbors), financial resources, and professional assistance.

A sample of responses (approximately 10%) was reviewed independently by two researchers to develop a keyword-driven coding frame. In general, there was a high level of concordance between the two independently developed coding frames. As there was no predetermined number of categories agreed before coding, the only aspect that required further agreement was the overall number of categories to code and analyze. This led to the merging of some small coding groups to form a "miscellaneous" category (a total of 0.4% of responses), and there was further separation of the single positive/active lifestyle category to form a positive lifestyle category and a sport and physical activity category. This resulted in identification of 17 content-specific, mutually exclusive categories, and the full set of responses was then coded into these categories. As respondents could provide a number of different comments, their responses were assigned to as many categories as required, typically one to three categories.

### Additional measures

As the target question was included as part of a larger survey, it was possible to investigate differences in the patterns of responses for different subgroups in the population. For the analysis presented here, we focused on four potentially vulnerable subgroups: women, those with low household incomes (<AUD$40,000), those with high psychological distress, and those who speak a language other than English at home.

Demographic questions relating to gender and income were used to identify the first two subgroups. For the present analysis, those with a household income of below AUD$40,000 were distinguished from those with an income of AUD$40,000 or greater. Socioeconomic factors are known to be related to a wide range of health and other vulnerabilities. Also, in the context of the current global financial crisis, it was felt that it would be interesting to investigate the strategies employed by less affluent individuals to see if differences existed and to try to understand how patterns of resilience in the population might be impacted by loss of income or reduction in financial resources.

Psychological distress was assessed using the Kessler 10 (K10) measure. The K10 provides a composite measure of nonspecific psychological distress, largely anxiety and depressive symptoms. The K10 has been used extensively in Australian population health research, with baseline data available at both state and national levels [11,12]. The K10 provides a score from 10 to 50. These scores are categorized as low (10-15), moderate (16-21), high (22-29), and very high (30-50). K10 categories have been linked to intervention strategies, and high levels have been shown to be associated with high probability of a mental disorder [13,14]. In data collected in NSW during 2007, prevalence estimates for high and very high psychological distress were 9.0% and 3.1%, respectively. In the present analysis, the categories of high and very high psychological distress have been combined.

The language other than English subgroup was selected for further investigation in the current analysis as previous research has identified differences in threat perceptions and responses by this group to specific threats of pandemic influenza and terrorism [8,9,15]. It was considered important to assess whether differences would be noted in the more generalized context of tough times. A single question on the main population health survey was used to identify this subgroup: "Do you usually speak a language other than English at home?"

### Data Analysis

Survey data were weighted to adjust for probability of selection and for differing response rates among males and females and different age groups. Data were manipulated and analyzed using Stata V9.2, which allowed for adjustments for sampling weights. Prevalence estimates and 95% confidence bands were calculated for each category coded. Statistical analysis of differences among these estimates for subgroups in the population was tested using Χ^2 ^tests.

## Results

Data were collected from 3,995 respondents during the period Jan. 22 to June 30, 2007. During this time, the reported response rate of the NSW Population Health Survey was 65%.

Responses to the question "What are the things that get you through tough times?" were coded into 17 categories, as previously described; 14 were the main subject-area categories, and the three remaining categories were "don't know" (9.0%), "refused" (0.2%), and "miscellaneous" (0.4%). The latter category contained diverse responses provided by very small numbers of respondents, and as such was not included in the developed coding frame.

Data were weighted, and prevalence estimates for each category of comment were calculated for the whole population and the four subpopulations. Of the weighted sample, 50.2% were female, 34.2% had household income less than AUD$40,000, 11.8% had high psychological distress, and 17.2% spoke a language other than English at home.

Table 1 summarizes the prevalence estimates for each category of comment for the 14 main subject-area categories. The prevalence estimates were calculated as the percentage of the population reporting in each category. As more than one category could be recorded for each respondent, the column percentages total more than 100%.

**Table 1:** Coding categories for responses to the question: "What are the things that get you through tough times?"

CATEGORY	EXPANDED DESCRIPTION	%	95% CI
Family and self	me, my spouse/partner, parents, children	51.7	(49.5 - 53.9)
Friends and neighbors	my friends, neighbors	21.0	(19.2 - 22.8)
Emotional and philosophical	positive thinking, determination, hopefulness, love, sense of humor, belief that things will get better, others worse off	17.4	(16.0 - 18.9)
Religious and spiritual	my belief/faith, prayer, spirituality	10.6	(9.4 - 11.9)
Leisure interests and hobbies	TV, music, gardening, fishing, reading, personal computer	6.3	(5.2 - 7.5)
Positive lifestyle	good health/staying healthy, sleep, time to self, relaxation, rest, winding-down time, holidays, work/life balance	3.9	(3.1 - 4.8)
Wider social support	community/social groups, nonspecific people	3.5	(2.8 - 4.3)
Practical	pragmatism, breaking things down, previous experience, training, planning, analysis, information, and advice	3.4	(2.6 - 4.4)
Sport and physical activity	specific sports, walking, being active	2.7	(2.1 - 3.6)
Drinking and smoking	alcohol, tobacco	2.1	(1.5 - 2.8)
Pets	my dog, cat, animals	0.8	(0.5 - 1.3)
Financial	money, financial security	0.8	(0.4 - 1.4)
Professional help	medical professionals, G.P., psychologist, counselor	0.6	(0.3 - 0.9)
Medication	medication, drugs, specific drugs	0.2	(0.1 - 0.4)

As can be seen from Table 1, the main resources and strategies employed to get through tough times were social supports, followed by emotional and philosophical strategies and religious and spiritual beliefs. More than half the population (51.7%) reported relying on family and self, followed by just over one-fifth (20.1%) relying on friends and neighbors. A wide range of emotional and philosophical strategies were also employed, with many people (17.4%) using optimism and positive thinking to move forward, while others considered those who are less well off or having a harder time as a way of putting their own problems into perspective. Religious and spiritual beliefs were identified by many as central to their ability to get through and recover from tough times (10.6%). Beyond these four most frequently reported strategies, a range of activities, behaviors, and practical and professional resources were employed by the general population. Practical, action-focused strategies were reported by 3.4% of the population, and professional help and support were mentioned by 0.6%. Health-related approaches through a focus on a positive lifestyle and through sports and physical activities were reported by 3.9% and 2.7%, respectively, and a less healthy, symptom-related strategy of drinking and smoking was reported by 2.1%.

To compare relative differences in the response profiles of subgroups in the population, Tables 2 and 3 provide prevalence estimates for males and females, those with household incomes less than and greater than AUD$40,000, those with high and low psychological distress, and those who do and do not speak a language other than English at home, respectively.

**Table 2:** Comparison of responses by gender and household income level.

Coping Strategies	Gender				Income					
	
	Male		Female			<AUD$40 k		>AUD$40 k		
	%	95% CI	%	95% CI	P values	%	95% CI	%	95% CI	P values
Family and self	49.7	(46.3-53.2)	53.7	(51.0-56.4)	0.075	39.8	(36.3-43.3)	60.1	(57.0-63.2)	**<0.001**
Friends and neighbours	17.0	(14.5-19.9)	24.9	(22.6-27.4)	**<0.001**	15.9	(13.5-18.7)	23.7	(21.1-26.5)	**<0.001**
Emotional and philosophical	18.5	(16.3-21.1)	16.2	(14.6-18.2)	0.118	22.6	(20.2-25.2)	16.0	(13.9-18.3)	**<0.001**
Religious and spiritual	7.3	(5.8-9.3)	13.8	(12.2-15.6)	**<0.001**	14.9	(12.8-17.4)	8.9	(7.3-10.8)	**<0.001**
Leisure interests and hobbies	6.5	(5.1-8.4)	6.0	(4.7-7.7)	0.645	6.7	(5.0-8.9)	5.6	(4.3-7.3)	0.364
Positive lifestyle	3.6	(2.4-5.3)	4.1	(3.2-5.2)	0.536	3.5	(2.5-4.9)	4.6	(3.4-6.2)	0.242
Wider social support	2.9	(2.0-4.3)	3.9	(3.1-5.0)	0.182	3.7	(2.7-4.9)	3.1	(2.2-4.2)	0.422
Practical	3.9	(2.7-5.9)	2.8	(2.1-3.7)	0.146	3.1	(2.3-4.2)	3.9	(2.7-5.6)	0.339
Sport and physical activity	3.3	(2.2-4.8)	2.2	(1.5-3.1)	0.131	1.7	(1.1-2.6)	3.5	(2.5-4.9)	**0.009**
Drinking and smoking	2.9	(1.9-4.2)	1.3	(0.8-2.1)	**0.011**	1.7	(0.9-3.4)	2.4	(1.7-3.5)	0.387
Pets	0.5	(0.1-1.7)	1.1	(0.7-1.7)	0.266	1.1	(0.4-3.0)	0.7	(0.4-1.2)	0.366
Financial	1.3	(0.6-2.5)	0.3	(0.1-0.8)	**0.011**	0.5	(0.2-1.1)	0.8	(0.4-1.8)	0.299
Professional help	0.4	(0.1-0.9)	0.8	(0.4-1.4)	0.191	0.9	(0.4-1.9)	0.6	(0.3-1.2)	0.460
Medication	0.2	(0.1-0.6)	0.2	(0.1-0.6)	0.945	0.3	(0.1-0.6)	0.3	(0.1-0.7)	0.743

**Table 3:** Comparison of responses by psychological distress level and language other than English spoken at home.

Coping Strategies	Psychological distress				Speak language other than English					
	
	Low		High			No		Yes		
	%	95% CI	%	95% CI	P values	%	95% CI	%	95% CI	P values
Family and self	53.2	(50.1-56.2)	55.9	(47.9-63.7)	0.527	53.7	(51.4-55.9)	42.7	(36.8-48.9)	**<0.001**
Friends and neighbours	22.7	(20.2-25.5)	21.6	(15.5-29.2)	0.771	22.1	(20.2-24.2)	15.8	(11.8-20.9)	**0.025**
Emotional and philosophical	17.8	(15.7-19.9)	12.7	(8.8-17.9)	0.071	18.7	(17.1-20.4)	11.0	(8.1-14.9)	**<0.001**
Religious and spiritual	10.7	(9.0-12.8)	10.9	(7.3-16.0)	0.934	10.2	(9.1-11.5)	12.2	(8.7-16.7)	0.340
Leisure interests and hobbies	6.3	(4.9-7.9)	12.9	(7.9-20.3)	**0.007**	5.7	(4.7-6.9)	8.8	(5.8-13.3)	0.066
Positive lifestyle	4.5	(3.4-6.1)	0.3	(0.1-1.2)	**<0.001**	4.3	(3.4-5.4)	1.8	(0.6-4.7)	0.073
Wider social support	3.6	(2.6-4.9)	2.6	(1.2-5.7)	0.456	3.5	(2.8-4.4)	3.1	(1.8-5.3)	0.644
Practical	3.0	(2.2-4.2)	3.1	(1.3-7.5)	0.944	3.3	(2.5-4.4)	3.7	(2.1-6.5)	0.749
Sport and physical activity	2.6	(1.8-3.4)	5.3	(2.3-11.9)	0.122	3.0	(2.3-3.9)	1.4	(0.4-4.6)	0.202
Drinking and smoking	1.3	(0.8-2.0)	9.7	(5.3-17.3)	**<0.001**	2.3	(1.6-3.1)	1.2	(0.4-3.3)	0.226
Pets	0.9	(0.4-2.1)	1.7	(0.6-4.9)	0.406	0.9	(0.5-1.5)	0.3	(0.1-0.8)	**0.045**
Financial	1.0	(0.4-2.4)	0.1	(0.02-0.9)	**0.031**	0.9	(0.4-1.6)	0.5	(0.1-1.9)	0.472
Professional help	0.4	(0.18-1.1)	2.7	(1.1-6.8)	**0.002**	0.6	(0.3-1.0)	0.5	(0.1-1.7)	0.798
Medication	0.1	(0.02-0.4)	0.5	(0.1-2.3)	0.073	0.3	(0.2-0.5)	0.0	0	0.132

In Table 2, there are a number of statistically significant differences in the prevalence of reported strategies for dealing with tough times between men and women. Compared to men, women were much more likely to report friends and neighbors as sources of support (p < 0.001) and were more likely to report the importance of religious and spiritual beliefs (p < 0.001), with women's rates double that of men's (14% and 7%, respectively). Conversely, men were more likely than women to report use of drinking and smoking (p = 0.01) and the importance of financial security (p = 0.01) to get through tough times.

With regard to the effects of household income, those on lower incomes (<AUD$ 40,000) were significantly less likely than those on higher incomes to report using social supports, such as family and self and friends and neighbors to get through tough times (both p < 0.001). Similarly, the lower-income group was less likely to use sports and physical activity as a strategy (p = 0.009). Those on lower incomes were, however, more likely to report finding emotional and philosophical strategies and religious and spiritual beliefs helpful for getting through tough times (both p < 0.001).

Table 3 summarizes the responses of those with different levels of psychological distress and those who do and don't speak a language other than English at home. Those with high levels of psychological distress reported similar degrees of use of social support to those with low psychological distress. However, compared to those with low psychological distress, those with high levels of psychological distress were more likely to use leisure interests and hobbies, much more likely to report drinking and smoking, and more likely to use professional help to get through tough times (p = 0.007, p < 0.001, and p < 0.002, respectively). Those with low psychological distress were much more likely to report using positive lifestyle approaches and relying on financial security (p < 0.001 and p = 0.031, respectively) as ways to get through tough times than those with high psychological distress. There was also a suggestion that those with high psychological distress were less likely to employ positive emotional or philosophical strategies than those with low psychological distress. However, this difference narrowly missed reaching a level of statistical significance (p = 0.071).

Compared to those who speak English at home, those who speak a language other than English at home were found to be less likely to report using social support to get through tough times, both family and self (p < 0.001) and friends and neighbors (p = 0.025). They were also much less likely to report using emotional and philosophical approaches or to turn to their pets (p < 0.001, and p = 0.045, respectively). There was some evidence that those who speak a language other than English at home were more likely to use leisure interests and hobbies, but this difference did not reach a level of statistical significance (p = 0.066).

## Discussion

When asked the question "What are the things that get you through tough times?" the vast majority of respondents (>90%) were able to identify at least one strategy they found helpful. Many respondents identified the importance of key people or groups, such as family, friends, and neighbors, and to a lesser extent, the importance of wider social support, such as social groups and church groups. The finding that social support is fundamental to handling adversity effectively is widely acknowledged and supported in the research literature [2,3,6].

Women were significantly more likely to report turning to friends and neighbors to get through tough times. This has also been reported in prior studies [16,17]. However, respondents who had lower incomes and those who spoke a language other than English at home were significantly less likely to report turning to their family, friends, or neighbors for support. This suggests a lower resource base for these two subgroups. One possible explanation is that these groups may have generally fewer family members or friends available to turn to, possibly as a function of duration of residence in Australia for some respondents, or there may be genuine cultural differences in how people feel they should relate to others in tough times or be willing to report such reliance. Those on lower incomes are possibly already under a degree of financial adversity or constraints and may have reduced access to people or groups for a range of practical or financial reasons, such as the costs of travel or the cost of events or tools that bring such groups together, such as meals, parties, computers, and telephones. Such general constraints may make it more difficult to sustain and nurture close relationships. Whatever the exact reasons, it does appear that those with lower incomes and those who speak a language other than English use less, or have reduced levels of, support from family, friends, and neighbors in tough times.

Given the high levels of reliance on the support of others to get through tough times and the apparent awareness of this in the general population, it is easy to appreciate the vulnerability that may be felt through temporary or permanent loss of such support due to bereavement, arguments, relocation, job loss, or social dislocation. Such losses may also be caused by natural disasters or drought resulting in job losses and erosion of rural communities, or by economic adversities, such as the global financial crisis, resulting in job loss or financial insecurity. Potential global health threats, such as pandemic influenza or emerging infectious diseases, could also require the use of physical control measures, such as social distancing, home quarantine, and school and work closures, all resulting in disruption to social support networks at a time when they may be needed most.

Internal- and emotion-focused strategies were frequently reported, including positive emotional and philosophical strategies, such as feeling optimistic and thinking about other people being worse off or a general belief that things would get better. As mentioned earlier, the benefits of such approaches have been shown to have measurable psychological benefits [5,2], and there is evidence of improved resilience to adversity over time [18]. Those with low incomes were more likely to report using positive emotional and philosophical approaches to get through tough times. With our data indicating that this group reports lower use of, or possibly access to, social support, it is encouraging that this group has identified positive emotion as an effective way of coping with adversity.

The importance of religious and spiritual beliefs was also noted by many as providing a source of strength in tough times. This was particularly so for women and those on lower incomes. In early research, there has been a general finding that women display more signs of religiosity than men [19]. It is possible that these strategies may be resistant to the impacts of disaster and external threats, although they may be more prone to disruption through psychological ill health, depression, or grief. Bleich et al [3], in their study on the impacts of prolonged terrorism in the Israeli population, also found that those who used "faith in God" as a method of coping with terrorism had a greater likelihood of feeling depressed, although it is likely that the sustained nature of the threat in this context may be a factor.

In the general population, leisure pursuits and activities were regarded as helpful, with people reporting their reliance on sporting activities, exercise, hobbies, and interests to get through tough times. It is unclear how effective such avoidant strategies are likely to be in the longer term. Leisure activities and hobbies may provide helpful distraction and diversion and may be helpful in reducing stress and improving affect; however, it is unlikely that such strategies alter the source or course of tough times unless they are practical in nature or lead to a cognitively based solution to such problems. Interestingly, those on lower incomes were significantly less likely to report using sporting and physical activity as a strategy for getting through tough times. This finding could be due to the associated costs -- e.g., gym memberships or clothing/equipment -- as well as other forms of support needed to allow for such activities, such as child care and time.

Importantly, many people mentioned specific actions they take to give themselves strength, including finding time to unwind and relax, taking holidays, eating healthfully, and getting rest. Those with higher psychological distress were significantly less likely to report employing such techniques. Given that the K10 measure of psychological distress comprises elements of depression and anxiety, it is possible that those with high levels of distress may be unlikely to draw on internal resources required to take care of, prioritize, or value their own needs or well-being. Disaster research has found that loss of resources such as these, termed "energy" resources, are associated with higher levels of acute stress disorder and depression in disaster-affected populations [7]. Taken together, these findings reinforce the links between psychological distress and positive lifestyle strategies. The consequences for coping and resilience to adversity in this group are evident.

A surprisingly small proportion of respondents reported a practical or instrumental approach to tackling tough times, such as applying previous experience or training, seeking further information or advice, or breaking the problems down into smaller issues that could be managed. It is possible that the question and use of the term "tough times" was too general to evoke this category of response and did not convey a focus on practical approaches or solutions. In the context of disasters or adversity, practical, pragmatic approaches are helpful and widely used [3], both in gathering information and taking action but also in setting goals, looking toward the future, and having constructive things to do. Where such strategies may be compromised is in situations that appear too overwhelming or that require resources that are unavailable. However, such situations, at least in the context of natural disasters in Australia, are likely to be short-lived, with information and resources quickly mobilized.

Symptom-based coping strategies, such as drinking alcohol, smoking, and taking medication, were reported but by a generally small proportion of the population. Alcohol consumption and smoking were more likely to be reported by those with high psychological distress and by men. Although the benefits of a single beer or glass of wine as a way to relax or unwind might be seen as an effective and harmless quick fix to a tough day, increases in drinking alcohol or smoking would be harmful as longer-term strategies and would be unlikely to resolve tough times.

Finally, and of relevance in the current tough economic times, a small proportion of respondents reported the use of money or financial security as a means to get through tough times. This was reported significantly less frequently by women and those with high psychological distress. A possible implication of this finding is that men may be more vulnerable in the current global financial crisis if exposed to financial loss, and in the wider context of disaster and adversity, would similarly want to rely more on financial security that may be compromised.

### Limitations

Although the data presented in this paper are taken from a large representative sample of the NSW population, there are limitations to this study that should be considered. A single question provides a simple snapshot of key strategies reported by respondents during an interview. Participant responses may not have been exhaustive. Some respondents appeared to be able to report more strategies that get them through tough times, including women, respondents with higher incomes, respondents with high psychological distress, and respondents who did not speak a language other than English at home. These groups may have a greater variety of sources of support, or they may be better able to articulate their responses to the question.

The data reported here were provided through the use of an open-ended question, and therefore are open to errors of transcription and coding. Coding errors and subjectivity were reduced by the use of keywords and phrases and using two independent raters to code the comments. Transcription errors were possible but were likely to be low due to the high level of experience and proficiency of the CATI facility interviewers.

The question used in this research study was deliberately inclusive and general, and was intended to evoke a wide range of approaches to the generalized, but inherently meaningful, concept of "tough times." As such, the responses do not address a single specific problem or threat but convey a generalized approach or response to adversity. Equally, the question did not aim to quantify the relative effectiveness or efficiency of strategies, although the wording implied that the strategies mentioned by respondents would be regarded as effective and supportive of individual resilience. Finally, the question relied on respondents being able to declare their strategies. This appears to have been possible, as the vast majority of respondents were able to provide answers. However, 9% could not provide answers in the context of the interview, and therefore their contribution is absent.

Despite these limitations, this study has been able to collect meaningful and insightful data from a representative sample of the general population and enable an appreciation of differential use and reporting of strategies and supports used to get people in different subgroups in the population through tough times.

## Conclusions

In the broad context of tough times in Australia, more than 90% of our general population sample has been able to report at least one helpful strategy or source of support for getting through tough times. Such findings are encouraging, although not surprising, given the generally high resilience and strength of individuals.

Our data quantified the importance of social support. This finding serves to expose the vulnerability we have, individually and collectively, to disruption in our relationships and how large-scale threats, such as pandemics, natural disasters, and even cyber-terrorism, could impact individual and population resilience.

In addition, this study was able to identify statistical differences in the relative reporting of supports and strategies in certain subgroups of the population. Findings that have implications for these subgroups include: less reliance on social support and greater reliance on positive emotional and philosophical strategies in those on lower household incomes; greater reliance on smoking and drinking and financial security in men; and greater reliance on smoking and drinking for those with high psychological distress.

Although this study does not attempt to judge the relative effectiveness of the reported strategies used to tackle tough times, it does provide baseline information that can be used as a comparison to reported coping strategies when adversities or tough times occur. Knowledge of the strategies the population uses to tackle tough times is the first step toward identifying the most effective strategies, which should be a focus for future research. This would provide a starting point for possible population-based interventions designed to promote the use of effective coping strategies following adversity and provide a useful reference for those tasked with supporting individuals and families through a range of tough times.

## Competing interests

The authors declare that they have no competing interests.

## Authors' contributions

All authors contributed equally to this work. All authors have read and approved the final manuscript.
